# Guideline-directed medical therapy prescribing patterns and in-hospital outcomes among heart failure patients during COVID-19

**DOI:** 10.1016/j.ahjo.2024.100440

**Published:** 2024-08-02

**Authors:** Pratyaksh K. Srivastava, Alexandra M. Klomhaus, Asim Rafique, Pooja S. Desai, Lori B. Daniels, Clyde W. Yancy, Eric H. Yang, Gregg C. Fonarow, Rushi V. Parikh

**Affiliations:** a Division of Cardiology, Ronald Reagan UCLA Medical Center, Los Angeles, CA, United States of America; b Department of Medicine, Statistics Core, UCLA, Los Angeles, CA, United States of America; c Department of Cardiology, Smidt Heart Institute, Cedars-Sinai Medical Center, Los Angeles, CA, United States of America; d Division of Cardiovascular Medicine, UC San Diego, La Jolla, CA, United States of America; e Division of Cardiology, Northwestern University School of Medicine, Chicago, IL, United States of America

**Keywords:** Guideline-directed medical therapy, Heart failure with reduced ejection fraction, COVID-19

## Abstract

**Study objective:**

The association of prior to admission guideline-directed medical therapy (GDMT) use in patients hospitalized with Heart Failure with Reduced Ejection Fraction (HFrEF, ejection fraction ≤40 %) and Coronavirus Disease 2019 (COVID-19) with in-hospital outcomes has not been well studied.

**Design/setting/participants/interventions/outcome measures:**

Using the American Heart Association's Get With The Guidelines Heart Failure Registry, we identified HFrEF patients presenting with acute decompensated heart failure (ADHF) and compared rates of GDMT prescription between those presenting prior to and during the pandemic. In a subgroup of patients with a concomitant COVID-19 diagnosis, we evaluated the association of prior to admission GDMT use with in-hospital mortality and severe COVID-19.

**Results:**

23,899 patients were admitted with HFrEF during the pandemic (2/16/20–3/24/21) and 26,459 patients were admitted in the year prior (2/16/19–2/15/20). In this overall cohort, prior to admission ACEI/ARB/ARNI (45.6 % vs 48.1 %, p < 0.0001) and BB (56.9 % vs 62.4 %, p < 0.0001) use was lower among admitted HFrEF patients during the pandemic when compared to the year prior. Rates of ACEI/ARB/ARNI, MRA, and triple therapy (ACE/ARB/ARNI + BB + MRA) prescription at discharge were higher during the pandemic compared to the year prior. Among a subgroup of those with HFrEF and COVID-19 (n = 333), prior to admission GDMT use was not associated with in-hospital mortality or severe COVID-19.

**Conclusion:**

We found no association between prior to admission GDMT use and in-hospital mortality or severe COVID-19 among HFrEF patients admitted with ADHF and COVID-19. GDMT prescription at discharge for HFrEF patients overall has remained either similar or improved during the pandemic.

## 1 Introduction

Heart failure is a common diagnosis in the United States, with an estimated prevalence of around 6.7 million among adults ≥20 years of age [1]. Heart failure with reduced ejection fraction (HFrEF) is a subset of heart failure defined by a left ventricular ejection fraction (LVEF) of ≤40 %. Large randomized controlled trials have elucidated a number of drug classes that significantly improve mortality in patients with HFrEF [[2], [3], [4], [5], [6]]. These agents, collectively termed guideline-directed medical therapy (GDMT), include 1) angiotensin converting enzyme inhibitors (ACEI)/angiotensin II receptor blockers (ARB)/angiotensin receptor-neprilysin inhibitors (ARNI), 2) beta blockers (BB), 3) mineralocorticoid receptor antagonists (MRA), and 4) sodium-glucose cotransporter 2 inhibitors (SGLT2i).

In January 2020, a novel coronavirus named Severe Acute Respiratory Syndrome Coronavirus 2 (SARS-CoV-2) was found to cause the clinical syndrome now known as Coronavirus Disease 2019 (COVID-19) [7]. Patients with pre-existing HFrEF experience increased morbidity and mortality from SARS-CoV-2, which mechanistically enters the human cell through viral spike protein binding to human angiotensin converting enzyme 2 (ACE2) [[8], [9], [10], [11], [12]]. Given a number of HFrEF therapeutics target the renin-angiotensin-aldosterone system (RAAS), there has been considerable interest in evaluating the safety of GDMT in HFrEF patients with COVID-19.

While several large studies have demonstrated the safety of RAAS inhibition [13,14] and beta blockade [15] in those from the general population with COVID-19, there are no studies to date focused on evaluating the impact of these agents in a HFrEF subset. To address this evidence gap, we evaluate the impact of prior to admission GDMT use on outcomes among hospitalized HFrEF patients with COVID-19 using the American Heart Association's Get With The Guidelines® Heart Failure (AHA GWTG-HF) registry. We also evaluate GDMT prescription rates during COVID-19, and compare them to a period prior to the pandemic.

## 2 Methods

### 2.1 Study population, definitions, and outcomes

The AHA GWTG-HF registry is a national quality improvement registry of patients hospitalized with acute decompensated heart failure (ADHF). Study protocols for the registry were approved by institutional review boards (IRB) at each site, and details of the registry have been previously described [16]. Each participating hospital received either human research approval to enroll cases without individual patient consent under the common rule, or a waiver of authorization and exemption from subsequent review by their IRB. Advarra, the IRB for the American Heart Association, determined that this study is exempt from IRB oversight. After excluding those with missing medication data, we identified an overall cohort of patients who were admitted with HFrEF during the pandemic (2/16/20–3/24/21) and in the year prior (2/16/19–2/15/20). Of those admitted during the pandemic, we also identified a subgroup of patients with a concomitant diagnosis of COVID-19. The medication intake form contained a selection box indicating if a patient was not on prior medical therapy; therefore, we were able to distinguish patients not on any medications prior to admission from those whose sites did not input medication data. The primary outcome was in-hospital mortality. The secondary outcome was severe COVID-19, which was defined as either use of mechanical ventilation, new dialysis, or in-patient mortality during admission. COVID-19 diagnosis was defined as active infection on admission or at some point during hospitalization. Triple therapy was defined as the combination of ACEI/ARB/ARNI, BB, and MRA use. Of note, SGLT2i were excluded from this analysis as this drug class was not routinely recorded in the AHA GWTG-HF registry during the study time period.

### 2.2 Statistical analysis

Patients in the subgroup (HFrEF and COVID-19) were subdivided based on prior GDMT status. Demographics, medical comorbidities, medical devices/prior procedures, and discharge disposition were compared between groups. Missingness of the cohort is shown in Supplemental Table 1. Continuous and categorical variables were compared using Wilcoxon Rank-Sum and Chi-Square tests, respectively. Continuous data are presented as median (25th–75th percentile) and categorical data presented as frequency (%). Next, using logistic regression, we evaluated the association of prior to admission GDMT use (individually and triple therapy) with in-hospital mortality, and with severe COVID-19. Logistic regression models were adjusted for age, sex, race, medical comorbidities (atrial fibrillation/atrial flutter, cerebrovascular accident, chronic kidney disease, coronary artery disease, diabetes mellitus, hypertension, hyperlipidemia, valvular heart disease and smoking in the last twelve months). Body mass index was not included in the initial models given its high rate of missingness (missing in 78/333 patients). Models involving ACEI, ARB, or ARNI were further adjusted for prior to admission BB and MRA use. The BB models were further adjusted for prior to admission ACEI/ARB/ARNI and MRA use, and the MRA models were adjusted for ACEI/ARB/ARNI and BB use. Logistic regression data are presented as odds ratio (95 % confidence interval). In a sensitivity analysis, the regression models above were repeated with the inclusion of body mass index.

To evaluate GDMT prescribing patterns, we compared prior to admission GDMT use, GDMT continuation during hospitalization, GDMT initiation at hospitalization or at discharge, and GDMT prescription at discharge in the overall cohort in those who presented prior to (2/16/19–2/15/20) and during the pandemic (2/16/20–3/24/21). Ineligible patients, defined as those with a contraindication to GDMT, were excluded where appropriate. For example, patients with a contraindication to a BB were excluded from the BB analysis only. For the ACEI/ARB groups, patients were excluded if they had a contraindication to both ACEI and ARBs. For the ACEI/ARB/ARNI group, patients were excluded if they had a contraindication to ACEI, ARB, and ARNIs. In categories involving discharge prescription, ineligible patients were additionally defined as those who died prior to discharge. Last, we compared reasons for GDMT non-prescription between the groups. Patients with contraindications to GDMT were included in the non-prescription analysis. All comparisons were made using Chi Square tests.

Statistical analyses were performed using SAS on the American Heart Association's Precision Medicine Platform [17]. The threshold for significance was set at a two-sided p-value of <0.05.

## 3 Results

### 3.1 Study population

**Fig. 1:**
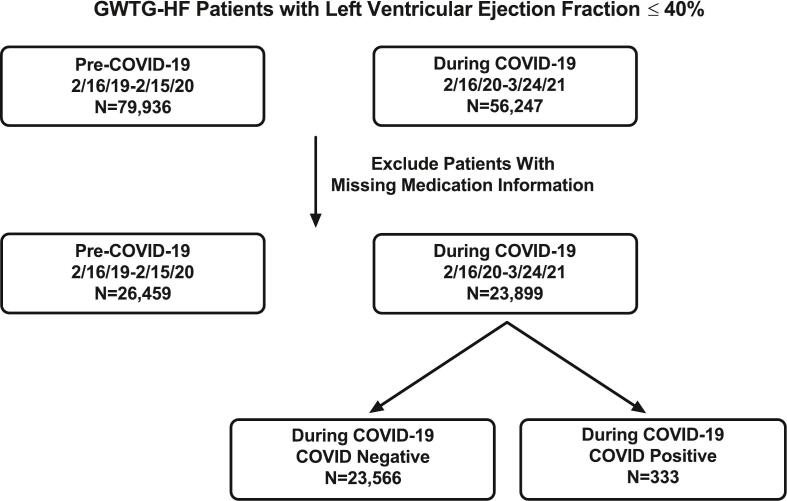
Population derivation. Abbreviations: COVID-19, Coronavirus Disease 2019; GWTG-HF, Get With the Guidelines Heart Failure.

The overall cohort consisted of 23,899 HFrEF patients who presented with ADHF during COVID-19 (2/16/20–3/24/21) and 26,459 HFrEF patients who presented with ADHF in the year prior to COVID-19 (2/16/19–2/15/20). Characteristics of the cohort are shown in Supplemental Table 2. The 23,899 HFrEF patients who presented with ADHF during the COVID-19 pandemic were further subdivided into those with (N = 333) and without (N = 23,566) a diagnosis of COVID-19 (Fig. 1). The baseline characteristics of those with HFrEF and COVID-19 (N = 333) stratified by GDMT status are shown in Table 1. 12.9 % of those with HFrEF and COVID-19 (43/333) had severe COVID-19. The median age was 65 (56–76) years, and 33.9 % of the cohort was female. Compared with patients on ACEI/ARB/ARNI prior to admission, patients not on ACEI/ARB/ARNI were more likely to have chronic kidney disease (CKD) including need for dialysis, diabetes mellitus, and prior myocardial infarction. Patients not on prior to admission beta blockers were less likely to have atrial fibrillation/flutter, more likely to have diabetes, and less likely to have smoked tobacco in the past 12 months compared to those on prior to admission beta blockers. Compared to patients on prior to admission MRA, patients not on MRA were older, more likely to have CKD, less likely to have depression, and more likely to have diabetes mellitus. Race, BMI, payment source, and discharge disposition did not significantly differ by prior to admission GDMT use (Table 1).

**Table 1:** Baseline characteristics of patients with HFrEF and COVID-19 stratified by prior to admission guideline-directed therapy use.

	Overall cohort	Prior ACEI/ARB/ARNI	No Prior ACEi/ARB/ARNI	p-Value	Prior evidence-based beta blocker	No prior evidence-based beta blocker	p-Value	Prior MRA	No prior MRA	p-Value
	n = 333	n = 143	n = 190		n = 189	n = 144		n = 56	n = 277	
Demographics^a^										
Age, years	65.0 (56.0–76.0)	64.0 (55.0–74.0)	67.5 (57.0–78.0)	0.10	65.0 (56.0–79.0)	65.0 (56.0–75.0)	0.65	61.5 (51.5–71.0)	67.0 (58.0–77.0)	0.01
Female, n (%)	113 (33.9)	50 (35.0)	63 (33.2)	0.73	64 (33.9)	49 (34.0)	0.97	21 (37.5)	92 (33.2)	0.54
Body mass index, kg/m^2	28.3 (24.4–32.9)	29.1 (24.6–34.2)	27.6 (24.3–32.4)	0.23	28.6 (24.5–33.3)	27.1 (24.0–32.1)	0.19	28.7 (24.8–36.4)	28.3 (24.2–32.6)	0.23
Race, n (%)				0.56						0.08
American Indian/Alaska Native	3 (0.9)	2 (1.4)	1 (0.5)		2 (1.1)	1 (0.7)	0.22	2 (3.6)	1 (0.4)	
Asian	10 (3.0)	5 (3.5)	5 (2.6)		2 (1.1)	8 (5.6)		3 (5.4)	7 (2.5)	
Native Hawaiian or Pacific Islander	2 (0.6)	1 (0.7)	1 (0.5)		1 (0.5)	1 (0.7)		1 (1.8)	1 (0.4)	
Black or African American	105 (31.5)	51 (35.7)	54 (28.4)		60 (31.8)	45 (31.3)		15 (26.8)	90 (32.5)	
White	184 (55.3)	74 (51.8)	110 (57.9)		109 (57.7)	75 (52.1)		32 (57.1)	152 (54.9)	
Other	29 (8.7)	10 (7.0)	19 (10.0)		15 (7.9)	14 (9.7)		3 (5.4)	26 (9.4)	
Ethnicity, n (%)										
Hispanic	67 (20.1)	23 (16.1)	44 (23.2)	0.11	33 (17.5)	34 (23.6)	0.17	12 (21.4)	55 (19.9)	0.79
Payment source, n (%)				0.48			0.21			0.26
Medicare	121 (44.0)	61 (51.7)	73 (46.5)		70 (45.2)	51 (42.5)		21 (40.4)	100 (44.8)	
Medicaid	63 (22.9)	39 (33.1)	35 (22.3)		38 (24.5)	25 (20.8)		18 (34.6)	45 (20.2)	
Private/HMO/PPO/other	59 (21.5)	27 (22.9)	32 (20.4)		29 (18.7)	30 (25.0)		8 (15.4)	51 (22.9)	
Veterans Affairs/CHAMP/Tricare	11 (4.0)	5 (4.2)	6 (3.8)		9 (5.8)	2 (1.7)		1 (1.9)	10 (4.5)	
Self-pay/no insurance	18 (6.6)	7 (5.9)	11 (7.0)		7 (4.5)	11 (9.2)		3 (5.8)	15 (6.7)	
Not documented	3 (1.1)	3 (2.5)	0 (0.0)		2 (1.3)	1 (0.8)		1 (1.9)	2 (0.9)	
Medical comorbidities, n (%)										
Anemia	99 (30.3)	36 (25.5)	63 (33.9)	0.10	50 (26.7)	49 (35.0)	0.11	16 (28.6)	83 (30.6)	0.76
Atrial fibrillation/flutter	117 (35.8)	51 (36.2)	66 (35.5)	0.90	80 (42.8)	37 (26.4)	0.00	18 (32.1)	99 (36.5)	0.53
Cerebrovascular accident/transient ischemic attack	49 (15.0)	19 (13.5)	30 (16.1)	0.51	29 (15.5)	20 (14.3)	0.76	10 (17.9)	39 (14.4)	0.51
Chronic kidney disease (serum creatine > 2.0 mg/dL)	81 (24.8)	22 (15.6)	59 (31.7)	<0.001	42 (22.5)	39 (27.9)	0.26	5 (8.9)	76 (28.0)	0.003
Chronic kidney disease on dialysis	19 (5.8)	3 (2.1)	16 (8.6)	0.01	8 (4.3)	11 (7.9)	0.17	0 (0.0)	19 (7.0)	0.05
Chronic obstructive pulmonary disease/asthma	101 (30.9)	51 (36.2)	50 (26.9)	0.07	63 (33.7)	38 (27.1)	0.20	15 (26.8)	86 (31.7)	0.47
Coronary artery disease	174 (53.2)	70 (49.7)	104 (55.9)	0.26	101 (54.0)	73 (52.1)	0.74	24 (42.9)	150 (55.4)	0.09
Depression	56 (17.1)	26 (18.4)	30 (16.1)	0.58	32 (17.1)	24 (17.1)	0.99	15 (26.8)	41 (15.1)	0.04
Diabetes mellitus	184 (56.3)	65 (46.1)	119 (64.0)	0.001	93 (49.7)	91 (65.0)	0.01	24 (42.9)	160 (59.0)	0.03
Hyperlipidemia	208 (63.6)	89 (63.1)	119 (64.0)	0.87	120 (64.2)	88 (62.9)	0.81	39 (69.6)	169 (62.4)	0.30
Hypertension	271 (82.9)	116 (82.3)	155 (83.3)	0.80	157 (84.0)	114 (81.4)	0.55	48 (85.7)	223 (82.3)	0.54
Peripheral vascular disease	33 (10.1)	11 (7.8)	22 (11.8)	0.23	19 (10.2)	14 (10.0)	0.96	3 (5.4)	30 (11.1)	0.20
Prior myocardial infarction	102 (31.2)	33 (23.4)	69 (37.1)	0.01	55 (29.4)	47 (33.6)	0.42	20 (35.7)	82 (30.3)	0.42
Smoking in last 12 months	72 (21.6)	36 (25.2)	36 (19.0)	0.17	51 (27.0)	21 (14.6)	0.01	14 (25.0)	58 (20.9)	0.50
Sleep disordered breathing	50 (15.3)	24 (17.0)	26 (14.0)	0.45	32 (17.1)	18 (12.9)	0.29	11 (19.6)	39 (14.4)	0.32
Valvular heart disease	73 (22.3)	32 (22.7)	41 (22.0)	0.89	45 (24.1)	28 (20.0)	0.38	15 (26.8)	58 (21.4)	0.38
Laboratory values										
Admission sodium, mmol/L	137 (134–140)	137 (134–140)	137 (134–140)	0.57	137 (134–140)	137 (134–140)	0.49	136.5 (133–140)	137 (134–140)	0.53
Admission potassium, mmol/L	4.2 (3.8–4.7)	4.2 (3.8–4.7)	4.2 (3.8–4.8)	0.56	4.2 (3.8–4.7)	4.2 (3.8–4.8)	0.82	4.3 (3.7–4.8)	4.2 (3.8–4.7)	0.94
Admission creatinine, mg/dL	1.3 (1.0–2.0)	1.3 (1.0–1.7)	1.3 (1.0–2.3)	0.13	1.3 (1.0–2.0)	1.3 (1.0–2.0)	0.27	1.3 (1.1–1.6)	1.3 (1.0–2.0)	0.68
Admission BUN, mg/dL	26.0 (17.0–40.0)	22.0 (17.0–37.0)	28.0 (18.0–45.0)	0.09	27.0 (18.0–42.0)	22.0 (16.0–35.0)	0.07	26.0 (19.0–38.0)	25.0 (17.0–42.0)	0.58
Medical devices/prior procedures, n (%)										
Coronary artery bypass graft	63 (19.3)	25 (17.7)	38 (20.4)	0.54	35 (18.7)	28 (20.0)	0.77	8 (14.3)	55 (20.3)	0.30
Cardiomems	2 (0.6)	0 (0.0)	2 (1.1)	0.22	0 (0.0)	2 (1.4)	0.18	0 (0.0)	2 (0.7)	1.00
Implantable cardioverter defibrillator/pacemaker/cardiac resynchronization therapy-defibrillator or pacemaker	104 (31.8)	41 (29.1)	63 (33.9)	0.36	57 (30.5)	47 (33.6)	0.55	23 (41.1)	81 (29.9)	0.10
Percutaneous coronary intervention	72 (22.0)	26 (18.4)	46 (24.7)	0.17	42 (22.5)	30 (21.4)	0.82	11 (19.6)	61 (22.5)	0.64
Discharge disposition, n (%)				0.13			0.91			0.61
Home	215 (64.6)	98 (68.5)	117 (61.6)		122 (64.6)	93 (64.6)		41 (73.2)	174 (62.8)	
Hospice (home or healthcare facility)	15 (4.5)	4 (2.8)	11 (5.8)		8 (4.2)	7 (4.9)		1 (1.8)	14 (5.1)	
Acute care facility or other healthcare facility	57 (17.1)	19 (13.3)	38 (20.0)		35 (18.5)	22 (15.3)		8 (14.3)	49 (17.7)	
Expired	32 (9.6)	13 (9.1)	19 (10.0)		17 (9.0)	15 (10.4)		4 (7.1)	28 (10.1)	
Other	14 (4.2)	9 (6.3)	5 (2.6)		7 (3.7)	7 (4.9)		2 (3.6)	12 (4.3)	

Abbreviations: ACEI, angiotensin converting enzyme inhibitor; ARB, angiotensin receptor II blocker; ARNI, angiotensin receptor-neprilysin inhibitor; CHAMP, Civilian Health and Medical Program; COVID-19, Coronavirus Disease 2019; HFrEF, heart failure with reduced ejection fraction; HMO, Health Maintenance Organization; MRA, mineralocorticoid receptor antagonist; PPO, Preferred Provider Organization.

a Continuous variables presented as median (25th–75th percentile). Continuous and categorical variables compared using Wilcoxon Rank-Sum Test, and Chi-Square tests, respectively.

### 3.2 Prior to admission GDMT use and in-hospital outcomes

Table 2 evaluates the impact of prior to admission GDMT use on in-hospital mortality and severe COVID-19. Prior to admission GDMT use alone (ACEI, ARB, ARNI, ACEI/ARB/ARNI, BB, MRA) or in combination (triple therapy: ACEI/ARB/ARNI + BB + AA) was not significantly associated with odds of in-hospital mortality or odds of severe COVID-19 in both unadjusted and adjusted logistic regression models. In sensitivity analysis adding body mass index to the models, prior to admission GDMT use alone or in combination still did not significantly associate with odds of in-hospital mortality or odds of severe COVID-19 (Supplemental Table 3).

**Table 2:** Association of prior to admission guideline-directed medical therapy with in-hospital outcomes among patients with heart failure with reduced ejection fraction and COVID-19.

	Unadjusted^a^			Adjusted^a^, ^b^		
	N outcome not on med prior to admission/N outcome on med prior to admission	OR (95 % CI)	P-value	N outcome not on med prior to admission/N outcome on med prior to admission	OR (95 % CI)	P-value
*Outcome: In-hospital mortality*						
Angiotensin converting enzyme inhibitor	**28/4**	0.49 (0.17, 1.44)	0.20	**28/4**	0.60 (0.19, 1.89)	0.38
Angiotensin II receptor blocker	**27/5**	1.41 (0.51, 3.89)	0.51	**27/5**	1.45 (0.49, 4.29)	0.51
Angiotensin receptor-neprilysin inhibitor	**28/4**	1.45 (0.47–4.44)	0.52	**28/4**	2.34 (0.63, 8.65)	0.20
ACEI/ARB/ARNI	**19/13**	0.90 (0.43–1.89)	0.78	**19/13**	1.24 (0.52, 2.97)	0.63
Beta blocker	**15/17**	0.85 (0.41–1.77)	0.66	**15/17**	0.82 (0.35, 1.94)	0.65
Mineralocorticoid receptor antagonist	**28/4**	0.68 (0.23–2.03)	0.49	**28/4**	0.78 (0.23, 2.63)	0.69
Triple therapy (ACEi/ARB/ARNI + BB + MRA)	**30/2**	0.68 (0.23–2.03)	0.49	**30/2**	0.63 (0.13, 2.98)	0.56

*Outcome: Severe disease*						
Angiotensin converting enzyme inhibitor	**37/6**	0.55 (0.22, 1.36)	0.20	**37/6**	0.61 (0.23, 1.61)	0.32
Angiotensin II receptor blocker	**38/5**	0.96 (0.35, 2.60)	0.93	**38/5**	0.90 (0.32, 2.57)	0.85
Angiotensin receptor neprilysin inhibitor	**38/5**	1.34 (0.48, 3.69)	0.58	**38/5**	2.16 (0.67, 6.93)	0.19
ACEI/ARB/ARNI	**27/16**	0.76 (0.39, 1.47)	0.42	**27/16**	0.90 (0.42, 1.95)	0.79
Beta-blocker	**20/23**	0.86 (0.45, 1.63)	0.64	**20/23**	0.98 (0.46, 2.08)	0.95
Mineralocorticoid receptor antagonist	**38/5**	0.62 (0.23, 1.64)	0.33	**38/5**	0.57 (0.19, 1.68)	0.31
Triple therapy (ACEi/ARB/ARNI + BB + MRA)	**40/3**	0.63 (0.18, 2.15)	0.46	**40/3**	0.55 (0.15, 2.01)	0.36

Abbreviations: ACEI, angiotensin converting enzyme inhibitor; ARB, angiotensin receptor II blocker; ARNI, angiotensin receptor-neprilysin inhibitor; COVID-19, Coronavirus Disease 2019; MRA, mineralocorticoid receptor antagonist.

a Logistic regression models compare those on prior to admission guideline-directed medical therapy medication to those who are not. Significance defined as p<0.05.

b Models adjusted for age, sex, race, medical comorbidities, and medications prior to admission.

### 3.3 GDMT prescription patterns

**Fig. 2:**
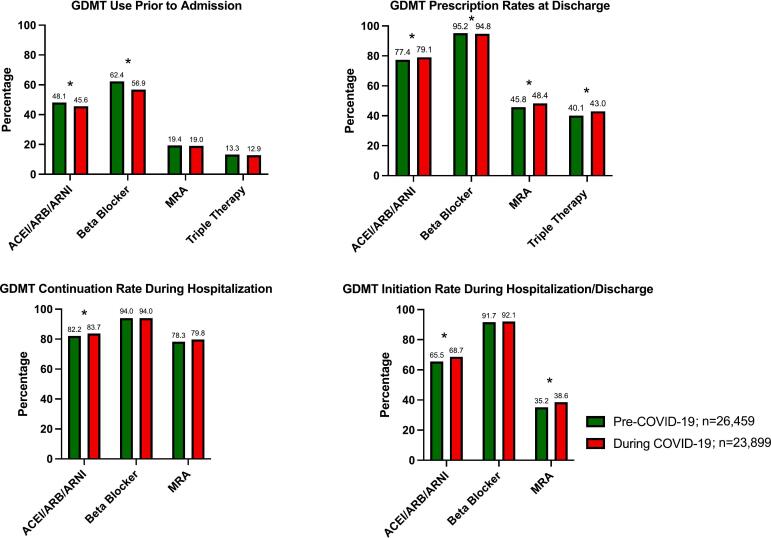
Guideline-directed medical therapy prescribing patterns pre- (2/16/19–2/15/20) and during (2/16/20–3/24/21) COVID-19. *Denotes statistically significant difference. Abbreviations: ACEI, angiotensin converting enzyme inhibitor; ARB, angiotensin receptor II blocker; ARNI, angiotensin receptor-neprilysin inhibitor; COVID-19, Coronavirus Disease 2019; MRA, mineralocorticoid receptor antagonist.

GDMT prescription patterns among eligible HFrEF patients pre- and during the COVID-19 pandemic are shown in Fig. 2 and Supplemental Table 4. Fewer patients were on ACEI/ARB/ARNI (45.6 % vs 48.1 %, p < 0.0001) and BB (56.9 % vs 62.4 %, p < 0.0001) prior to admission during COVID-19 when compared to the year prior. There were no differences in prior to admission MRA or triple therapy between the two time periods. ACEI/ARB/ARNI (83.7 % vs 82.2 %, p = 0.01) were continued during hospitalization more during COVID-19 compared to the year prior. There were no differences in frequency of BB and MRA continuation. ACEI/ARB/ARNI (68.7 % vs 65.5 %, p < 0.0001) and MRA (38.6 % vs 35.2 %, p < 0.0001) were started more frequently during the pandemic among those who were not on these respective therapies prior to admission compared to the year prior. ACEI/ARB/ARNI (79.1 % vs 77.4 %, p < 0.0001), MRA (48.4 % vs 45.8 %, p < 0.0001), and triple therapy (43.0 % vs 40.1 %, p < 0.0001) were prescribed at discharge more during the pandemic compared to the year prior while BB were prescribed slightly less (94.8 % vs 95.2 %, p = 0.03). When looking at ARNI use alone, ARNI was used more prior to admission, continued more during hospitalization, started more during hospitalization or at discharge, and prescribed more at discharge in the during COVID-19 time period compared to the pre-COVID-19 time period (all p values <0.05, Supplemental Table 4).

**Fig. 3:**
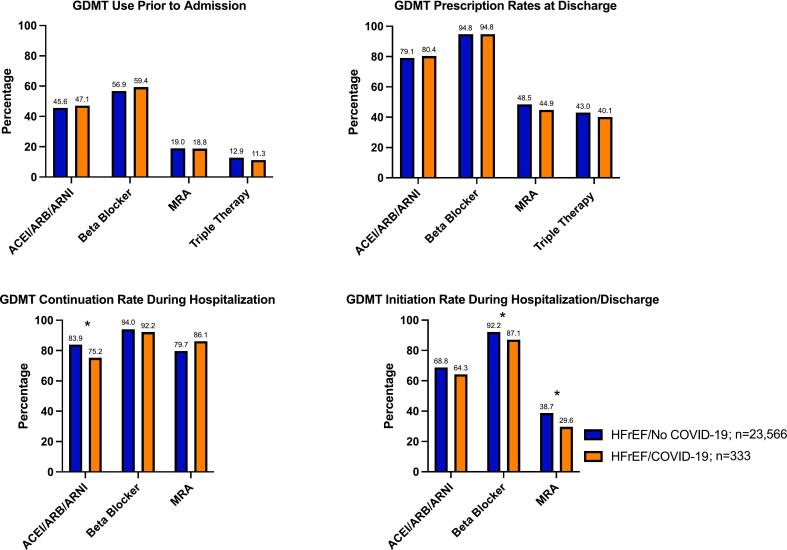
Guideline-directed medical therapy prescribing patterns during (2/16/20–3/24/21) the COVID-19 pandemic among HFrEF patients with and without COVID-19. *Denotes statistically significant difference. Abbreviations: ACEI, angiotensin converting enzyme inhibitor; ARB, angiotensin receptor II blocker; ARNI, angiotensin receptor-neprilysin inhibitor; COVID-19, Coronavirus Disease 2019; MRA, mineralocorticoid receptor antagonist.

GDMT prescription patterns among eligible HFrEF patients during the COVID-19 pandemic stratified by COVID-19 status are shown in Fig. 3 and Supplemental Table 4. During the COVID-19 time period, there were no differences in rates of prior to admission GDMT when comparing HFrEF patients with COVID-19 to those without. HFrEF patients with COVID-19 were less frequently continued on ACEI/ARB/ARNI (75.2 % vs 83.9 %, p = 0.01) compared with HFrEF patients without COVID-19. There were no differences in BB and MRA continuation rates between the two groups. Among HFrEF patients not on GDMT prior to admission, patients with COVID-19 were less frequently initiated on BB (87.1 % vs 92.2 %, p = 0.04) and MRA (29.6 % vs 38.7 %, p = 0.01) during hospitalization/at discharge compared to patients without COVID-19. Last, there were no significant differences in GDMT prescription at discharge when comparing the two groups.

### 3.4 Reasons for GDMT non-prescription

Reasons for GDMT non-prescription are shown in Supplemental Table 5. For ACEI/ARB, marked azotemia (17.6 % vs 21.0 %, p < 0.0001) was marked as a reason for non-prescription less during the COVID-19 pandemic and system reason (1.6 % vs 0.8 %, p < 0.0001) was marked as a reason more during the pandemic compared to the year prior. There were no significant differences in reasons for ACE/ARB non-prescription during the pandemic when stratified by COVID-19 infection status. For ARNI, hyperkalemia (2.7 % vs 2.2 %, p = 0.01) and hypotension (14.4 % vs 13.3 %, p = 0.03) were marked as reasons for non-prescription more frequently during the pandemic, and ACEi use within the prior 36 h (29.7 % vs 36.1 %, p < 0.0001) and renal dysfunction (23.4 % vs 24.6 %, p = 0.04) were marked as reasons for non-prescription less frequently during the pandemic when compared to the year prior. For BB, fluid overload was marked as a reason for non-prescription less during the pandemic compared to the year prior (4.1 % vs 5.5 %, p = 0.02). For MRAs, renal dysfunction was more frequently marked as a reason for non-prescription (43.3 % vs 33.1 %, p = 0.03) when comparing those with HFrEF and COVID-19 to those with HFrEF and no COVID-19 during the pandemic period.

## 4 Discussion

In this national AHA GWTG registry-based analysis of HFrEF patients hospitalized with ADHF and COVID-19, we found that prior to admission GDMT use alone (ACEI, ARB, ARNI, ACEI/ARB/ARNI, BB, AA) or in combination as triple therapy (ACEI/ARB/ARNI + BB + AA) was not significantly associated with in-hospital mortality or severe COVID-19. Additionally, GDMT prescription at discharge during the pandemic among eligible HFrEF patients, irrespective of COVID-19 infection status, remained similar to or better than the year prior. Taken together, these data demonstrate for the first time, to our knowledge, that prior to admission GDMT use does not appear to associate with in-hospital mortality or odds of developing severe COVID-19 among admitted HFrEF patients with COVID-19. Furthermore, they highlight that GDMT prescription patterns at discharge have remained relatively intact during the COVID-19 pandemic.

### 4.1 SARS-CoV-2 and the renin-angiotensin-aldosterone system

SARS-CoV-2 enters human cells through viral spike protein binding to human ACE2 [8,9]. There has been considerable interest in the safety of heart failure therapeutics in patients with COVID-19 given their interaction with RAAS pathways. Specifically, concerns were expressed early in the pandemic that drugs such as ACEI/ARB and MRA may increase ACE2 expression, and therefore may increase susceptibility to SARS-CoV-2 [18]. These concerns have led to a number of studies that have evaluated the safety and impact of RAAS inhibition in patients with COVID-19. In a population of 12,594 patients from a large academic medical center, there was no association between prior to admission ACEI, ARB, or BB use and likelihood of a positive COVID-19 test [13]. In a separate cohort of 8.28 million patients, prior to admission ACEI/ARB use was associated with reduced risk of a COVID-19 positive test and was not associated with risk of receiving intensive care unit care among those infected with COVID-19 [19]. Among patients hospitalized with COVID-19 who were previously taking ACEI/ARBs, there was no significant difference in days alive out of the hospital at 30 days or COVID-19 severity when comparing those who discontinued the drugs during hospitalization to those who did not [20,21]. In a randomized clinical trial of 679 critically ill patients with COVID-19, however, initiation of ACE or ARB did not improve and likely worsened clinical outcomes leading to cessation of enrollment [22]. With regards to MRAs, in a study of nearly 1.4 million patients, and in a subsequent meta-analysis of nearly 1.39 million patients, MRA use was not associated with mortality from COVID-19 [23]. Similar studies have also demonstrated the safety of BB in those with COVID-19 [15,24].

### 4.2 Heart failure therapeutics and COVID-19

While the aforementioned studies have evaluated RAAS inhibition and beta blockade in large, general populations with COVID-19, there have been no studies to date evaluating the safety of these therapeutics in a HFrEF-specific cohort. Herein, we found no significant association between prior to admission ACEI/ARB/ARNI, BB, and MRA use and in-hospital mortality or severe COVID-19 among HFrEF patients admitted with COVID-19. There are a number of reasons why RAAS inhibition may not produce adverse outcomes in those with COVID-19. First, there is limited direct evidence that ACEI/ARBs significantly increase ACE2 expression on the surface of human cells. If we extrapolate from animal models and assume increased ACE2 expression, it is still unknown how much the expression is augmented or how quickly the ACE2 expression will decrease after cessation of ACEI/ARBs [[25], [26], [27], [28], [29]]. Second, it is unclear that increased cellular ACE2 expression translates into increased SARS-CoV-2 binding and susceptibility. In fact, ACEI/ARBs may prove to be beneficial in those who are subsequently infected with SARS-CoV-2 by preventing binding and internalization of SARS-CoV-2. In a mouse study by Deshotels et al., ACE2 and the receptor for Angiotensin II (AT1R) were found to form complexes that were reduced by treatment with Ang II [30]. Further, treatment with Ang II enhanced ACE2 ubiquitination and internalization [30]. The study also demonstrated that the ARB losartan prevented ACE2 internalization and degradation [30]. Based on these findings, Sparks et al. have hypothesized that the ACE2-AT1R complex may stabilize ACE2 in low Ang II states (ie. treatment with ACEI/ARB), and may possibly diminish SARS-CoV-2 binding and internalization [31]. While the majority of trials surrounding RAASi have suggested no signal of harm, one randomized clinical trial among 679 critically ill COVID-19 patients suggested worse hospital survival among patients randomized to start an ACEI or an ARB [22]. Of note, patients were defined as critically ill if they were in an intensive care unit receiving vasopressors/inotropes or significant respiratory support (high flow nasal cannula with flow rate ≥ 30 L/min or noninvasive/invasive mechanical ventilation). This degree of respiratory and cardiovascular illness in these patients likely contributed significantly to their relative intolerance of RAASi and overall worse outcomes.

### 4.3 GDMT prescription patterns during COVID-19

In this study, we also evaluated GDMT prescription patterns during the pandemic. While ACEI/ARB/ARNI and BB use prior to admission were lower during the pandemic when compared to the year prior, ACEI/ARB/ARNI initiation during hospitalization, continuation during hospitalization, and prescription at discharge were all higher during the pandemic when compared to the year prior. Rates of GDMT prescription (ACEI/ARB/ARNI, MRA, triple therapy) at discharge in general remained similar or higher during the pandemic compared to the year prior with the exception of BB prescription, which was lower during COVID-19 compared to the year prior (94.8 % vs 95.2 %, p = 0.03). This absolute difference between the groups is very small, however, and unlikely to be clinically significant. Comparing HFrEF patients with COVID-19 to those without COVID-19 during the pandemic period, there were similar rates of prior to admission GDMT use, though ACEI/ARB/ARNI were continued less frequently in those with COVID-19. This finding is likely related to increased rates of acute kidney injury and lower blood pressures often seen during hospitalization in COVID-19 patients [32]. Reassuringly, GDMT prescription rates at discharge remained similar between HFrEF patients with and without COVID-19. A study by Keshvani et al. also analyzed GDMT prescription rates at discharge in GWTG-HF, and demonstrated slightly higher percentages of GDMT prescription at discharge when compared to our findings [33]. The authors also demonstrated lower rates of MRA and ACE/ARB/ARNI prescription at discharge among those with HFrEF and COVID-19 compared to those with HFrEF and no COVID-19 [33]. These differences in findings are likely attributable to differences in population definition and sample size.

### 4.4 Limitations

This study has some limitations worth considering. Data for this study were collected retrospectively and therefore causation cannot be assumed. While logistic regression models were adjusted for patient demographics and characteristics, the chance for residual confounding remains. While similar months were used to compare the pre-COVID 19 with the during COVID 19 population, these groups still came from different time periods, which may add additional confounding. The overall cohort of patients with COVID-19 and HFrEF was small (N = 333), and so this analysis would benefit from being repeated once larger numbers are available. Data were obtained from patients enrolled in the GWTG-HF registry, and therefore may not be fully generalizable to the overall population. Patients with entirely missing medication sections were excluded from the analysis, which may further limit generalizability. COVID-19 specific therapies were not evaluated. While SGLT2i play an important role in the management of HFrEF patients, they were excluded from this analysis as data on this therapeutic drug class was not routinely collected in GWTG-HF during the study time period. Finally, given registry design, assessment of long-term outcomes beyond the patient's hospitalization was not possible.

## 5 Conclusion

In this national AHA GWTG registry-based study of HFrEF patients admitted with ADHF and COVID-19, we found no significant association between prior to admission GDMT use alone or in combination with in-hospital mortality or severe COVID-19. In addition, GDMT prescription patterns at discharge have remained either similar or improved during the pandemic among eligible HFrEF patients, regardless of COVID-19 status.

## Financial support

The Get With The Guidelines®–Heart Failure (GWTG-HF) program is provided by the 10.13039/100000968American Heart Association. GWTG-HF is sponsored, in part, by 10.13039/100004336Novartis, 10.13039/100001003Boehringer Ingelheim, 10.13039/501100004191Novo Nordisk, 10.13039/100004325AstraZeneca, 10.13039/100004326Bayer, Tylenol and 10.13039/100006400Alnylam Pharmaceuticals.

## Ethics statement

All procedures were performed in compliance with relevant laws and institutional guidelines and were approved by the appropriate institutional committee(s). Advarra, the IRB for the American Heart Association, determined that this study is exempt from IRB oversight.

## CRediT authorship contribution statement

**Pratyaksh K. Srivastava:** Writing – review & editing, Writing – original draft, Visualization, Validation, Supervision, Software, Resources, Project administration, Methodology, Investigation, Formal analysis, Conceptualization. **Alexandra M. Klomhaus:** Writing – review & editing, Writing – original draft, Formal analysis, Conceptualization. **Asim Rafique:** Writing – review & editing, Writing – original draft, Conceptualization. **Pooja S. Desai:** Writing – review & editing, Writing – original draft, Conceptualization. **Lori B. Daniels:** Writing – review & editing, Writing – original draft. **Clyde W. Yancy:** Writing – review & editing, Writing – original draft, Supervision. **Eric H. Yang:** Writing – review & editing, Writing – original draft, Supervision, Resources, Conceptualization. **Gregg C. Fonarow:** Writing – review & editing, Writing – original draft, Resources, Conceptualization. **Rushi V. Parikh:** Writing – review & editing, Writing – original draft, Validation, Supervision, Software, Methodology, Formal analysis, Conceptualization.

## Declaration of competing interest

The authors declare the following financial interests/personal relationships which may be considered as potential competing interests: GCF reports consulting for Abbott, Amgen, AstraZeneca, Bayer, Cytokinetics, Edwards, Eli Lilly, Johnson&Johnson, Medtronic, Merck, Novartis, and Pfizer.

EHY reports research grants/funding from CSL Behring, Boehringer Ingelheim, Eli Lilly, and Bristol Meyers Squibb, and consulting fees from Pfizer.

LBD reports consulting for Abbott, Quidel, and Roche; research funding from Vifor; and serves on a clinical endpoint adjudication committee for Applied Therapeutics.

RVP receives unrelated research support from Infraredx, Abbott Vascular, and Bayer, and consulting fees from Abbott Vascular.

## Appendix A Supplementary data

Supplementary tables Image 1
